# *trans*-(Pyrimidine-2-thiol­ato-κ^2^ *N*,*S*)[tris­(2-amino­ethyl)amine-κ^4^ *N*,*N*′,*N*′′,*N*′′′]cobalt(III) chloride hexa­fluoridophosphate

**DOI:** 10.1107/S1600536809032395

**Published:** 2009-08-22

**Authors:** Keisuke Fujihara, Toshiaki Yonemura

**Affiliations:** a Department of Applied Science, Faculty of Science, Kochi University, Akebono-cho, Kochi 780-8520, Japan

## Abstract

In the title compound, [Co(C_4_H_3_N_2_S)(C_6_H_18_N_4_)](Cl)PF_6_, the Co^III^ ion is coordinated by a tripod-like tetra­dentate ligand and a monoanionic *N*,*S*-bidentate ligand in an approximately octa­hedral CoN_4_OS geometry. The anionic S atom of the pyrimidine-2-thiol­ate (pymt) ligand is coordinated in the *trans* position to the primary amine N atom (Nprim) of the tris­(2-amino­ethyl)amine (tren) ligand. The crystal structure exhibits short inter­molecular N—H⋯N hydrogen bonds (N⋯N <3.2 Å), and inter­molecular N—H⋯Cl and C—H⋯F contacts, leading to the formation of an infinite two-dimensional network.

## Related literature

For the synthesis and chemistry of similar tren [tren = tris­(2-amino­ethyl)amine] complexes, see: Jackson & Sargeson (1978 ▶); Kojima *et al.* (1994 ▶); Mitsui *et al.* (1976 ▶); Ohba & Saito (1984 ▶); Okamoto *et al.* (1990 ▶); Yonemura *et al.* (1997 ▶).
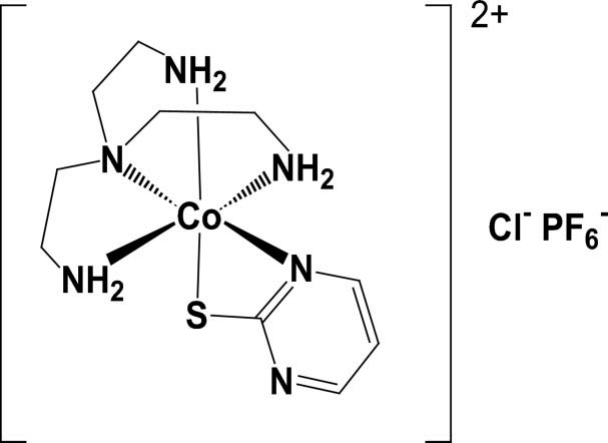


## Experimental

### 

#### Crystal data

[Co(C_4_H_3_N_2_S)(C_6_H_18_N_4_)](Cl)PF_6_
*M* *_r_* = 496.73
Monoclinic, 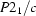

*a* = 12.7106 (17) Å
*b* = 11.326 (2) Å
*c* = 14.205 (2) Å
β = 112.549 (10)°
*V* = 1888.7 (5) Å^3^
*Z* = 4
Mo *K*α radiation
μ = 1.31 mm^−1^
*T* = 296 K
0.45 × 0.35 × 0.20 mm

#### Data collection

Rigaku AFC-7S diffractometer
Absorption correction: ψ scan (North *et al.*, 1968 ▶) *T*_min_ = 0.503, *T*_max_ = 0.770
5201 measured reflections
4342 independent reflections
3416 reflections with *F* ^2^ > 2σ(*F* ^2^)
*R*_int_ = 0.031
3 standard reflections every 150 reflections intensity decay: 5.4%

#### Refinement

*R*[*F* ^2^ > 2σ(*F* ^2^)] = 0.057
*wR*(*F* ^2^) = 0.174
*S* = 1.05
4342 reflections
237 parameters
H-atom parameters constrained
Δρ_max_ = 1.14 e Å^−3^
Δρ_min_ = −0.87 e Å^−3^

### 

Data collection: *WinAFC* (Rigaku/MSC, 2000 ▶); cell refinement: *WinAFC*; data reduction: *CrystalStructure* (Rigaku/MSC, 2007 ▶); program(s) used to solve structure: *SHELXS97* (Sheldrick, 2008 ▶); program(s) used to refine structure: *SHELXL97* (Sheldrick, 2008 ▶); molecular graphics: *ORTEP-3 for Windows* (Farrugia, 1997 ▶); software used to prepare material for publication: *CrystalStructure*.

## Supplementary Material

Crystal structure: contains datablocks global, I. DOI: 10.1107/S1600536809032395/su2138sup1.cif

Structure factors: contains datablocks I. DOI: 10.1107/S1600536809032395/su2138Isup2.hkl

Additional supplementary materials:  crystallographic information; 3D view; checkCIF report

## Figures and Tables

**Table 1:** Hydrogen-bond geometry (Å, °)

*D*—H⋯*A*	*D*—H	H⋯*A*	*D*⋯*A*	*D*—H⋯*A*
N4—H8⋯N2^i^	0.90	2.50	3.079 (4)	123
N4—H9⋯Cl1^ii^	0.90	2.52	3.278 (3)	142
N5—H14⋯Cl1^ii^	0.90	2.39	3.287 (3)	173
N5—H15⋯Cl1^iii^	0.90	2.42	3.273 (3)	160
N6—H21⋯Cl1	0.90	2.29	3.187 (3)	173
C6—H6⋯F1	0.97	2.39	3.210 (6)	143
C7—H11⋯F4^iv^	0.97	2.52	3.328 (6)	141
C9—H17⋯F6^v^	0.97	2.52	3.454 (6)	161

Symmetry codes: (i) 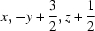; (ii) 
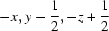
; (iii) 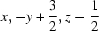; (iv) 
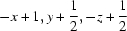
; (v) 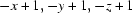.

**Fig. 1.**
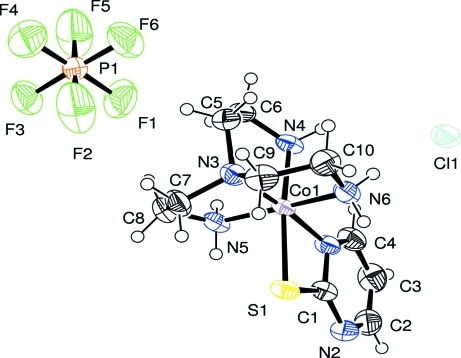
A view of the molecular structure of the title compound, showing the atom-labelling scheme and 50% probability displacement ellipsoids.

**Fig. 2.**
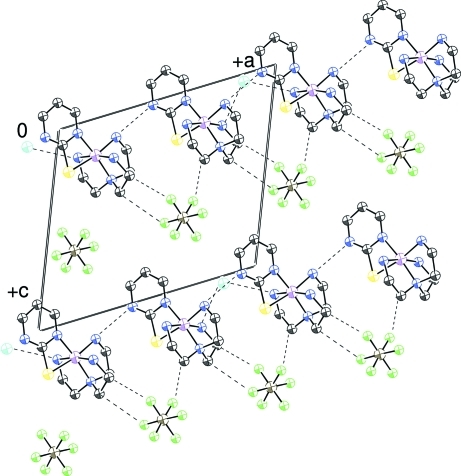
A view along the b-axis of the crystal packing diagram of the title compound, showing the N–H···N hydrogen-bonds and N–H···Cl and C–H···F contacts as dashed lines (the H-atoms have been omitted for clarity).

## supplementary crystallographic information

### Comment

Cobalt(III)-tren [tren = tris(2-aminoethyl)amine] complexes with thiolate and/or thioether ligands have been investigated in view of their stereochemistry and ligand substitution reactions (Mitsui & Kimura, *et al.*, 1976; Jackson & Sargeson *et al.*, 1978; Ohba & Saito, *et al.*, 1984; Okamoto, *et al.*, 1990; Kojima, *et al.*, 1994; Yonemura, *et al.*, 1997). The chemistry of the aliphatic and aromatic thiolato cobalt(III) complexes has led to many interesting results concerning the features of the coordinated sulfur atoms. Although aliphatic thiolato cobalt(III)-tren complexes have been extensively investigated, aromatic thiolato complexes have not been thus far because of the difficulties involved in their preparation.

In view of our interest in the stereochemistry and spectrochemical properties of aromatic and aliphatic cobalt(III) complexes, we synthesized the title compound using the tripod-like tetradentate tren ligand to fix the remaining two *cis* coordination sites in the cobalt(III) complexes, as two geometrical isomers, *p-*(*trans*(*Nprim,S*))and *t-*(*trans*(*Ntert,S*)), are possible for [Co(bidentate-*N,S*)(tren)]-type complexes. The known cobalt(III)-tren complexes of 2-aminoethanethiolate-*N,S *(aet) (Sargeson *et al.*, 1978) formed *p-*isomers as the major products, and those of 2-mercaptoacetate-*O,S* (ma) or 3-mercaptopropionate-*O,S* (mp) selectively formed the *t-*isomers (Mitsui & Kimura, *et al.*, 1976; Jackson & Sargeson *et al.*, 1978; Ohba & Saito, *et al.*, 1984; Okamoto, *et al.*,1990; Kojima, *et al.*, 1994; Yonemura, *et al.*, 1997). Herein we report on the structure of a cobalt(III)-tren complex coordinated with the aromatic thiolato ligand pyrimidine-2-thiolate (pymt).

In the title compound the cobalt(III) atom is coordinated by a tripod-like tetradentate ligand and a monoanionic N,*S*-bidentate ligand, producing an approximately octahedral CoN_4_OS geometry (Fig. 1). In the present complex the anionic sulfur atom of the pymt ligand is coordinated *trans* to the primary amine N-atom (Nprim) of the tren ligand, with a nearly linear S1—Co1—N4 angle (170.71 (9)Å) hence the title compound is the *p-*isomer (*trans*(*Nprim,S*)). The known cobalt(III)-tren complex of aet shows the same systematic trend, at least in the coordination manner of the *N,S*-bidentate ligand. The Co—S distance (2.3187 (12) Å) is significantly longer than those observed in the Co(III)-tren complexes with aliphatic thiolato ligands: 2.239 (1) Å in *t-*[Co{CH_3_SCH(CH_3_)COO}(tren)]^2+^ (Ohba & Saito, *et al.*, 1984), 2.236 (2) Å in *t-*[Co(mp)(tren)]^+^ and 2.232 (1) Å in *t-*[Co(ma)(tren)]^+^ (Yonemura, *et al.*, 1997)). The Co—N4 distance (1.954 (3) Å), which is in the *trans* position relative to the sulfur atom, is shorter than the other Co-N(tren) distances (Co1—N5 = 1.966 (3) Å and Co1—N6 = 1.970 (3) Å). The S1-Co1-N1 angle of 72.31 (9)° is far from the ideal angle of 90°. This distortion is relaxed by the other angles in the same plane, that is, S1-Co1-N3 = 101.96 (9), and N1-Co1-N4 = 98.40 (12)°. The other bond distances and angles are similar to those in the cobalt(III)-tren complexes mentioned above.

The aromatic pymt *N,S*-chelate ring is almost planar and the three *N,N'*-chelate rings of the tren are in a *gauche* conformation. The *gauche* conformations of the two –N—CH_2_—CH_2_—NH_2_ chelates in the tren ligand have unsymmetrical skew forms with the λ and δ conformations, and the *gauche* conformation of the central –N—CH_2_—CH_2_—NH_2_ chelate in the tren ligand has an unsymmetrical skew form with the λ conformation.

In the crystal structure each complex cation is linked to adjacent cations through N–H···N hydrogen bonds so constructing a one-dimensional chain structure (Fig. 2 and Table 1). These chains are further bridged by ordered Cl and PF_6_ anions through N–H···Cl and C–H···F contacts, leading to the formation of an infinite two-dimensional network (Table 1 and Fig. 2).

### Experimental

A methanolic solution of 2-pyrimidinethiol (1.12 g, 10 mmol) was adjusted to pH 8.0 with aqueous solutions of NaOH and HCl. The mixed solution was added to an aqueous solution of [CoCl_2_(tren)]Cl (3.11 g, 10 mmol) and stirred at 40 °C for 1 h. The reaction mixture was poured onto an SP-Sepahadex C-25 column (Na^+^ form, 7 cm × 7 cm) and the adsorbed band was developed with a 0.2 mol dm^-3^ NaCl aqueous solution. The eluted red band was concentrated to a small volume, the precipitated NaCl was filtered off and the red filtrate was added to a solution of NH_4_PF_6_ (1.1 g, 6.7 mmol). The resulting red powder was collected by filtration and recrystallized from water. A few days later red crystals of the title compoun were obtained with a yield of 1.50 g (35%). Anal. Calc. for [Co(pymt)(tren)]ClPF_6_ = C_10_H_21_CoN_6_SClPF_6_: C 24.18, H 4.26, N 16.92%, Found: C 24.09, H 4.24, N 17.04%.

### Refinement

The H-atoms were included in calculated positions and treated as riding atoms: N—H = 0.90 Å, C—H = 0.93–0.97 Å, with *U*_iso_(H) = 1.2*U*_eq_(C,*N*).

### Crystal data

**Table:**

[Co(C_4_H_3_N_2_S)(C_6_H_18_N_4_)](Cl)PF_6_	*F*(000) = 1008.0
*M**_r_* = 496.73	*D*_x_ = 1.747 Mg m^−^^3^
Monoclinic, *P*2_1_/*c*	Mo *K*α radiation, λ = 0.71069 Å
Hall symbol: -P 2ybc	Cell parameters from 25 reflections
*a* = 12.7106 (17) Å	θ = 15.4–17.1°
*b* = 11.326 (2) Å	µ = 1.31 mm^−^^1^
*c* = 14.205 (2) Å	*T* = 296 K
β = 112.549 (10)°	Prismatic, red-brown
*V* = 1888.7 (5) Å^3^	0.45 × 0.35 × 0.20 mm
*Z* = 4	

### Data collection

**Table:**

Rigaku AFC-7S diffractometer	*R*_int_ = 0.031
ω–2θ scans	θ_max_ = 27.5°
Absorption correction: ψ scan (North *et al.*, 1968)	*h* = −16→15
*T*_min_ = 0.503, *T*_max_ = 0.770	*k* = −14→0
5201 measured reflections	*l* = −10→18
4342 independent reflections	3 standard reflections every 150 reflections
3416 reflections with *F*^2^ > 2σ(*F*^2^)	intensity decay: 5.4%

### Refinement

**Table:**

Refinement on *F*^2^	H-atom parameters constrained
*R*[*F*^2^ > 2σ(*F*^2^)] = 0.057	*w* = 1/[σ^2^(*F*_o_^2^) + (0.1106*P*)^2^ + 1.816*P*] where *P* = (*F*_o_^2^ + 2*F*_c_^2^)/3
*wR*(*F*^2^) = 0.174	(Δ/σ)_max_ = 0.001
*S* = 1.05	Δρ_max_ = 1.14 e Å^−^^3^
4342 reflections	Δρ_min_ = −0.87 e Å^−^^3^
237 parameters	Extinction correction: *SHELXL97* (Sheldrick, 2008)
0 restraints	Extinction coefficient: 0.0077 (13)

### Special details

**Table:**

Geometry. Bond distances, angles etc. have been calculated using the rounded fractional coordinates. All su's are estimated from the variances of the (full) variance-covariance matrix. The cell esds are taken into account in the estimation of distances, angles and torsion angles
Refinement. Refinement was performed using all reflections. The weighted *R*-factor (*wR*) and goodness of fit (*S*) are based on *F*^2^. *R*-factor (gt) are based on *F*. The threshold expression of *F*^2^ > 2.0 σ(*F*^2^) is used only for calculating *R*-factor (gt).

### Fractional atomic coordinates and isotropic or equivalent isotropic displacement parameters (Å^2^)

**Table:**

	*x*	*y*	*z*	*U*_iso_*/*U*_eq_	
Co1	0.18778 (4)	0.70419 (4)	0.19018 (3)	0.0308 (1)	
S1	0.23158 (8)	0.80886 (9)	0.06968 (7)	0.0410 (3)	
N1	0.0476 (2)	0.7685 (3)	0.0930 (2)	0.0372 (9)	
N2	0.0184 (3)	0.8748 (3)	−0.0597 (2)	0.0470 (10)	
N3	0.3357 (2)	0.6418 (3)	0.2771 (2)	0.0385 (8)	
N4	0.1255 (2)	0.6241 (3)	0.2792 (2)	0.0406 (9)	
N5	0.1706 (2)	0.5600 (3)	0.1086 (2)	0.0405 (9)	
N6	0.2288 (2)	0.8465 (3)	0.2764 (2)	0.0410 (9)	
C1	0.0848 (3)	0.8230 (3)	0.0267 (2)	0.0384 (10)	
C2	−0.0930 (4)	0.8686 (4)	−0.0804 (3)	0.0548 (12)	
C3	−0.1387 (3)	0.8134 (4)	−0.0174 (3)	0.0568 (14)	
C4	−0.0639 (3)	0.7636 (4)	0.0716 (3)	0.0488 (12)	
C5	0.3252 (3)	0.5685 (3)	0.3615 (2)	0.0452 (11)	
C6	0.2038 (3)	0.5283 (3)	0.3347 (2)	0.0438 (11)	
C7	0.3755 (3)	0.5708 (4)	0.2084 (3)	0.0508 (12)	
C8	0.2801 (4)	0.4922 (4)	0.1429 (3)	0.0516 (12)	
C9	0.4102 (3)	0.7457 (4)	0.3219 (3)	0.0484 (11)	
C10	0.3430 (3)	0.8304 (3)	0.3597 (3)	0.0493 (12)	
P1	0.35869 (9)	0.19313 (10)	0.36387 (9)	0.0445 (3)	
F1	0.2435 (3)	0.2566 (4)	0.2930 (3)	0.1068 (16)	
F2	0.4274 (5)	0.2804 (3)	0.3247 (6)	0.142 (3)	
F3	0.3517 (3)	0.1030 (3)	0.2757 (2)	0.0873 (12)	
F4	0.4707 (3)	0.1283 (5)	0.4302 (3)	0.1148 (16)	
F5	0.2846 (5)	0.1060 (4)	0.3963 (5)	0.147 (3)	
F6	0.3610 (4)	0.2851 (4)	0.4487 (3)	0.1020 (16)	
Cl1	0.03840 (9)	0.91504 (11)	0.36135 (8)	0.0531 (3)	
H1	−0.14280	0.90310	−0.14030	0.0660*	
H2	−0.21700	0.81010	−0.03460	0.0680*	
H3	−0.09110	0.72690	0.11630	0.0590*	
H4	0.34970	0.61460	0.42370	0.0540*	
H5	0.37450	0.50000	0.37360	0.0540*	
H6	0.18900	0.45820	0.29230	0.0530*	
H7	0.19180	0.50880	0.39630	0.0530*	
H8	0.11730	0.67600	0.32400	0.0490*	
H9	0.05650	0.59400	0.24190	0.0490*	
H10	0.44060	0.52320	0.24880	0.0610*	
H11	0.39870	0.62300	0.16570	0.0610*	
H12	0.29470	0.46570	0.08420	0.0610*	
H13	0.27480	0.42330	0.18150	0.0610*	
H14	0.11560	0.51450	0.11480	0.0490*	
H15	0.14950	0.57950	0.04250	0.0490*	
H16	0.43190	0.78360	0.27070	0.0580*	
H17	0.47890	0.72090	0.37780	0.0580*	
H18	0.33480	0.79860	0.42000	0.0590*	
H19	0.38220	0.90560	0.37730	0.0590*	
H20	0.23020	0.90950	0.23830	0.0490*	
H21	0.17640	0.85960	0.30340	0.0490*	

### Atomic displacement parameters (Å^2^)

**Table:**

	*U*^11^	*U*^22^	*U*^33^	*U*^12^	*U*^13^	*U*^23^
Co1	0.0335 (2)	0.0386 (2)	0.0257 (2)	−0.0010 (2)	0.0173 (2)	0.0001 (2)
S1	0.0416 (4)	0.0527 (5)	0.0362 (4)	−0.0043 (3)	0.0232 (3)	0.0054 (3)
N1	0.0375 (14)	0.0471 (17)	0.0312 (14)	0.0010 (12)	0.0177 (11)	0.0025 (12)
N2	0.057 (2)	0.0517 (19)	0.0315 (14)	0.0037 (15)	0.0162 (14)	0.0037 (13)
N3	0.0372 (14)	0.0458 (16)	0.0349 (14)	0.0015 (12)	0.0164 (12)	0.0003 (13)
N4	0.0485 (17)	0.0501 (17)	0.0316 (14)	−0.0057 (13)	0.0246 (13)	−0.0012 (12)
N5	0.0490 (17)	0.0472 (17)	0.0318 (14)	−0.0058 (13)	0.0226 (13)	−0.0046 (12)
N6	0.0473 (17)	0.0433 (16)	0.0369 (15)	−0.0022 (13)	0.0211 (13)	−0.0040 (13)
C1	0.0427 (18)	0.0446 (19)	0.0325 (16)	−0.0017 (15)	0.0194 (14)	−0.0001 (14)
C2	0.057 (2)	0.058 (2)	0.041 (2)	0.012 (2)	0.0096 (18)	−0.0013 (19)
C3	0.037 (2)	0.070 (3)	0.058 (2)	0.0057 (19)	0.0121 (18)	−0.006 (2)
C4	0.044 (2)	0.063 (2)	0.046 (2)	0.0001 (18)	0.0245 (17)	−0.0018 (19)
C5	0.054 (2)	0.046 (2)	0.0344 (17)	0.0047 (17)	0.0156 (16)	0.0055 (15)
C6	0.059 (2)	0.044 (2)	0.0335 (16)	−0.0009 (17)	0.0235 (16)	0.0045 (14)
C7	0.047 (2)	0.066 (2)	0.046 (2)	0.0145 (19)	0.0253 (17)	−0.0033 (19)
C8	0.069 (2)	0.049 (2)	0.045 (2)	0.0116 (19)	0.031 (2)	−0.0071 (17)
C9	0.0367 (18)	0.058 (2)	0.045 (2)	−0.0066 (17)	0.0094 (15)	0.0031 (19)
C10	0.057 (2)	0.043 (2)	0.041 (2)	−0.0059 (17)	0.0112 (17)	−0.0054 (16)
P1	0.0437 (5)	0.0468 (5)	0.0452 (5)	0.0057 (4)	0.0196 (4)	0.0026 (4)
F1	0.084 (2)	0.113 (3)	0.102 (3)	0.046 (2)	0.012 (2)	0.005 (2)
F2	0.170 (5)	0.072 (2)	0.264 (7)	−0.021 (2)	0.171 (5)	−0.006 (3)
F3	0.103 (2)	0.085 (2)	0.073 (2)	0.016 (2)	0.0329 (19)	−0.0226 (18)
F4	0.089 (2)	0.148 (4)	0.077 (2)	0.048 (2)	−0.002 (2)	0.002 (2)
F5	0.182 (5)	0.078 (2)	0.265 (7)	0.003 (3)	0.180 (5)	0.022 (3)
F6	0.120 (3)	0.112 (3)	0.073 (2)	0.015 (2)	0.036 (2)	−0.027 (2)
Cl1	0.0510 (5)	0.0707 (7)	0.0425 (5)	0.0165 (4)	0.0235 (4)	0.0068 (4)

### Geometric parameters (Å, °)

**Table:**

Co1—S1	2.3187 (12)	N4—H9	0.9000
Co1—N1	1.930 (3)	N5—H15	0.9000
Co1—N3	1.944 (3)	N5—H14	0.9000
Co1—N4	1.954 (3)	N6—H21	0.9000
Co1—N5	1.966 (3)	N6—H20	0.9000
Co1—N6	1.970 (3)	C2—C3	1.389 (6)
Co1—C1	2.566 (3)	C3—C4	1.378 (6)
S1—C1	1.734 (4)	C5—C6	1.511 (5)
P1—F3	1.592 (3)	C7—C8	1.504 (6)
P1—F4	1.557 (5)	C9—C10	1.513 (6)
P1—F5	1.551 (6)	C2—H1	0.9300
P1—F6	1.585 (4)	C3—H2	0.9300
P1—F1	1.595 (4)	C4—H3	0.9300
P1—F2	1.556 (6)	C5—H5	0.9700
N1—C1	1.354 (4)	C5—H4	0.9700
N1—C4	1.332 (5)	C6—H6	0.9700
N2—C2	1.333 (7)	C6—H7	0.9700
N2—C1	1.330 (4)	C7—H10	0.9700
N3—C5	1.506 (4)	C7—H11	0.9700
N3—C7	1.495 (5)	C8—H13	0.9700
N3—C9	1.491 (5)	C8—H12	0.9700
N4—C6	1.479 (5)	C9—H16	0.9700
N5—C8	1.498 (6)	C9—H17	0.9700
N6—C10	1.491 (5)	C10—H19	0.9700
N4—H8	0.9000	C10—H18	0.9700
			
S1—Co1—N1	72.31 (9)	H14—N5—H15	108.00
S1—Co1—N3	101.96 (9)	Co1—N5—H14	109.00
S1—Co1—N4	170.71 (9)	H20—N6—H21	108.00
S1—Co1—N5	89.62 (9)	Co1—N6—H20	110.00
S1—Co1—N6	87.67 (9)	C10—N6—H21	110.00
S1—Co1—C1	41.18 (9)	Co1—N6—H21	110.00
N1—Co1—N3	173.92 (12)	C10—N6—H20	110.00
N1—Co1—N4	98.40 (12)	Co1—C1—N1	47.51 (15)
N1—Co1—N5	91.63 (13)	Co1—C1—S1	61.73 (10)
N1—Co1—N6	95.02 (13)	Co1—C1—N2	171.7 (3)
N1—Co1—C1	31.15 (12)	N1—C1—N2	125.2 (4)
N3—Co1—N4	87.33 (12)	S1—C1—N1	109.2 (2)
N3—Co1—N5	86.23 (13)	S1—C1—N2	125.6 (3)
N3—Co1—N6	86.65 (13)	N2—C2—C3	123.6 (4)
N3—Co1—C1	143.02 (12)	C2—C3—C4	117.6 (4)
N4—Co1—N5	90.85 (13)	N1—C4—C3	119.5 (4)
N4—Co1—N6	93.09 (13)	N3—C5—C6	111.1 (2)
N4—Co1—C1	129.54 (12)	N4—C6—C5	109.1 (3)
N5—Co1—N6	171.69 (12)	N3—C7—C8	109.1 (3)
N5—Co1—C1	89.96 (11)	N5—C8—C7	109.0 (3)
N6—Co1—C1	93.16 (11)	N3—C9—C10	107.4 (3)
Co1—S1—C1	77.09 (11)	N6—C10—C9	107.8 (3)
F5—P1—F6	91.6 (3)	N2—C2—H1	118.00
F1—P1—F2	89.6 (3)	C3—C2—H1	118.00
F1—P1—F3	91.8 (2)	C2—C3—H2	121.00
F1—P1—F4	178.1 (2)	C4—C3—H2	121.00
F1—P1—F5	87.7 (3)	N1—C4—H3	120.00
F1—P1—F6	85.9 (2)	C3—C4—H3	120.00
F2—P1—F3	89.8 (3)	N3—C5—H5	109.00
F2—P1—F4	90.4 (3)	N3—C5—H4	109.00
F2—P1—F5	176.5 (4)	H4—C5—H5	108.00
F2—P1—F6	90.3 (3)	C6—C5—H4	109.00
F3—P1—F4	86.3 (2)	C6—C5—H5	109.00
F3—P1—F5	88.2 (3)	N4—C6—H6	110.00
F3—P1—F6	177.7 (2)	C5—C6—H7	110.00
F4—P1—F5	92.2 (3)	N4—C6—H7	110.00
F4—P1—F6	96.0 (2)	C5—C6—H6	110.00
Co1—N1—C1	101.4 (2)	H6—C6—H7	108.00
Co1—N1—C4	139.2 (3)	H10—C7—H11	108.00
C1—N1—C4	119.0 (3)	N3—C7—H10	110.00
C1—N2—C2	115.1 (3)	N3—C7—H11	110.00
Co1—N3—C5	110.3 (2)	C8—C7—H10	110.00
Co1—N3—C7	105.5 (2)	C8—C7—H11	110.00
Co1—N3—C9	106.6 (2)	N5—C8—H12	110.00
C5—N3—C7	112.3 (3)	N5—C8—H13	110.00
C5—N3—C9	109.3 (3)	C7—C8—H12	110.00
C7—N3—C9	112.6 (3)	C7—C8—H13	110.00
Co1—N4—C6	109.2 (2)	H12—C8—H13	108.00
Co1—N5—C8	110.8 (2)	N3—C9—H16	110.00
Co1—N6—C10	109.8 (2)	N3—C9—H17	110.00
C6—N4—H9	110.00	C10—C9—H16	110.00
H8—N4—H9	108.00	C10—C9—H17	110.00
Co1—N4—H8	110.00	H16—C9—H17	108.00
C6—N4—H8	110.00	N6—C10—H18	110.00
Co1—N4—H9	110.00	N6—C10—H19	110.00
C8—N5—H14	110.00	C9—C10—H18	110.00
C8—N5—H15	109.00	C9—C10—H19	110.00
Co1—N5—H15	109.00	H18—C10—H19	108.00
			
N1—Co1—S1—C1	−1.46 (15)	N4—Co1—N6—C10	85.4 (2)
N3—Co1—S1—C1	176.44 (15)	C1—Co1—N6—C10	−144.7 (2)
N5—Co1—S1—C1	90.38 (14)	S1—Co1—C1—N1	−177.3 (3)
N6—Co1—S1—C1	−97.48 (14)	N1—Co1—C1—S1	177.3 (3)
S1—Co1—N1—C1	1.86 (19)	N3—Co1—C1—S1	−5.8 (2)
S1—Co1—N1—C4	173.9 (4)	N3—Co1—C1—N1	176.9 (2)
N4—Co1—N1—C1	−178.3 (2)	N4—Co1—C1—S1	179.46 (13)
N4—Co1—N1—C4	−6.3 (4)	N4—Co1—C1—N1	2.2 (3)
N5—Co1—N1—C1	−87.2 (2)	N5—Co1—C1—S1	−89.47 (12)
N5—Co1—N1—C4	84.8 (4)	N5—Co1—C1—N1	93.2 (2)
N6—Co1—N1—C1	87.8 (2)	N6—Co1—C1—S1	82.83 (12)
N6—Co1—N1—C4	−100.1 (4)	N6—Co1—C1—N1	−94.5 (2)
C1—Co1—N1—C4	172.1 (6)	Co1—S1—C1—N1	2.1 (2)
S1—Co1—N3—C5	−179.45 (19)	Co1—S1—C1—N2	−175.4 (3)
S1—Co1—N3—C7	−58.0 (2)	Co1—N1—C1—S1	−2.5 (3)
S1—Co1—N3—C9	62.0 (2)	Co1—N1—C1—N2	175.0 (3)
N4—Co1—N3—C5	0.4 (2)	C4—N1—C1—Co1	−174.1 (4)
N4—Co1—N3—C7	121.9 (3)	C4—N1—C1—S1	−176.6 (3)
N4—Co1—N3—C9	−118.2 (2)	C4—N1—C1—N2	0.9 (6)
N5—Co1—N3—C5	−90.6 (2)	Co1—N1—C4—C3	−170.8 (3)
N5—Co1—N3—C7	30.8 (2)	C1—N1—C4—C3	0.3 (6)
N5—Co1—N3—C9	150.8 (2)	C2—N2—C1—S1	175.8 (3)
N6—Co1—N3—C5	93.6 (2)	C2—N2—C1—N1	−1.2 (5)
N6—Co1—N3—C7	−144.9 (2)	C1—N2—C2—C3	0.4 (6)
N6—Co1—N3—C9	−24.9 (2)	Co1—N3—C5—C6	20.3 (3)
C1—Co1—N3—C5	−175.55 (18)	C7—N3—C5—C6	−97.1 (3)
C1—Co1—N3—C7	−54.1 (3)	C9—N3—C5—C6	137.2 (3)
C1—Co1—N3—C9	65.9 (3)	Co1—N3—C7—C8	−47.1 (4)
N1—Co1—N4—C6	156.7 (2)	C5—N3—C7—C8	73.1 (4)
N3—Co1—N4—C6	−21.3 (2)	C9—N3—C7—C8	−163.0 (3)
N5—Co1—N4—C6	64.9 (2)	Co1—N3—C9—C10	46.4 (3)
N6—Co1—N4—C6	−107.8 (2)	C5—N3—C9—C10	−72.8 (4)
C1—Co1—N4—C6	155.58 (18)	C7—N3—C9—C10	161.7 (3)
S1—Co1—N5—C8	93.0 (2)	Co1—N4—C6—C5	37.0 (3)
N1—Co1—N5—C8	165.3 (3)	Co1—N5—C8—C7	−15.2 (4)
N3—Co1—N5—C8	−9.0 (3)	Co1—N6—C10—C9	27.8 (3)
N4—Co1—N5—C8	−96.3 (3)	N2—C2—C3—C4	0.6 (7)
C1—Co1—N5—C8	134.2 (3)	C2—C3—C4—N1	−1.0 (6)
S1—Co1—N6—C10	−103.9 (2)	N3—C5—C6—N4	−37.6 (3)
N1—Co1—N6—C10	−175.9 (2)	N3—C7—C8—N5	40.9 (4)
N3—Co1—N6—C10	−1.8 (2)	N3—C9—C10—N6	−48.7 (4)

### Hydrogen-bond geometry (Å, °)

**Table:**

*D*—H···*A*	*D*—H	H···*A*	*D*···*A*	*D*—H···*A*
N4—H8···N2^i^	0.90	2.50	3.079 (4)	123
N4—H9···Cl1^ii^	0.90	2.52	3.278 (3)	142
N5—H14···Cl1^ii^	0.90	2.39	3.287 (3)	173
N5—H15···Cl1^iii^	0.90	2.42	3.273 (3)	160
N6—H21···Cl1	0.90	2.29	3.187 (3)	173
C6—H6···F1	0.97	2.39	3.210 (6)	143
C7—H11···F4^iv^	0.97	2.52	3.328 (6)	141
C9—H17···F6^v^	0.97	2.52	3.454 (6)	161

Symmetry codes: (i) *x*, −*y*+3/2, *z*+1/2; (ii) −*x*, *y*−1/2, −*z*+1/2; (iii) *x*, −*y*+3/2, *z*−1/2; (iv) −*x*+1, *y*+1/2, −*z*+1/2; (v) −*x*+1, −*y*+1, −*z*+1.
